# Poor quality of life and sleep in patients with adrenal insufficiency—another cause of increased mortality?

**DOI:** 10.1007/s11845-021-02731-y

**Published:** 2021-08-13

**Authors:** Antje K. Blacha, Peter Kropp, Amir H. Rahvar, Jörg Flitsch, Iris van de Loo, Birgit Harbeck

**Affiliations:** 1 I. Department of Medicine, University of Luebeck, Luebeck, Germany; 2 Institute of Medical Psychology and Medical Sociology, University of Rostock, Rostock, Germany; 3 III. Department of Medicine, University Medical Center Hamburg-Eppendorf, Hamburg, Germany; 4 Department of Neurosurgery, University Medical Center Hamburg-Eppendorf, Hamburg, Germany; 5 Diabetes-Schwerpunktpraxis Bremen, Bremen, Germany; 6 Amedes Experts, Hamburg, Germany

**Keywords:** Adrenal insufficiency, Hydrocortisone, PAI, Quality of life, SAI, Sleep

## Abstract

**Background:**

Patients with adrenal insufficiency (AI) are treated with glucocorticoid replacement therapy (GRT). Although current glucocorticoid regimens aim to mimic the physiological circadian rhythm of cortisol secretion, temporary phases of hypo- and hypercortisolism are common undesired effects which lead to a variety of consequences like increased cardiovascular risk and premature mortality. Additionally, poor quality of life (QoL) and impaired sleep have been reported. However, little is known about these topics regarding the effects of daily dosage, duration of therapy, and patients with different forms of AI (primary, PAI, and secondary, SAI).

**Methods:**

In this study, 40 adults with AI substituted with hydrocortisone (HC) and 20 matched healthy controls completed questionnaires evaluating depressive symptoms, subjective health status, quality of sleep and daytime sleepiness. Furthermore, demographic data, dosage of HC, duration of therapy and co-medication were evaluated. Patients were compared in different groups.

**Results:**

Patients assessed general health significantly worse than controls; likewise, daytime sleepiness was reported significantly more often. Depressive symptoms differed significantly in the two groups but did not reach clinically relevant scores. There was no difference between patients with PAI and SAI. High dosage of hydrocortisone had negative impact on mental health but not on sleep quality or daytime sleepiness.

**Conclusions:**

The present data highlight that poor QoL and impaired sleep are still severe and underrated issues in current GRT and might be additional factors for premature mortality in patients with AI. Some AI patients reach normal or near-normal self-assessed QoL and sleep, even despite unphysiological replacement.

## Introduction

Adrenal insufficiency (AI) is a rare endocrine disorder defined as the inability of the adrenal glands to achieve adequate hormonal production due to adrenal disease (primary AI, PAI) or due to malfunction of either the pituitary gland (secondary AI, SAI) or the hypothalamus (tertiary AI, TAI). All types of AI lead to a lack of endogenous glucocorticoids (GCs) which have to be substituted to prevent harmful symptoms that may even lead to death.

Current guidelines recommend 15–25 mg/d hydrocortisone (HC) for patients with PAI and 15–20 mg/d HC for patients with SAI [1, 2]. Despite splitting daily dosage into two or three portions throughout the day to mimic a physiological pattern, temporary phases of hyper- and hypocortisolism cannot be prevented [3]. Current literature reports a variety of consequences due to this unphysiological therapy: increased cardiovascular risk [4, 5], premature mortality [6, 7], and impaired cognitive functioning [8–10]. Restrictions in quality of life (QoL) and to a lesser degree in sleep are described as well [11–15]. 

Nevertheless, glucocorticoid replacement therapy (GRT) has remained almost unchanged in the past years. Co-medication with dehydroepiandrosteron (DHEA) may have positive effects on mood and fatigue [16, 17], but as yet, there are no hard criteria for use. Administration of dual-release HC preparations is a promising strategy to minimize problems of conventional GRT [18, 19].

The aim of this pilot study was to evaluate QoL and sleep in patients with AI in a complex approach. Both PAI and SAI patients were included with special focus on comparison of these patients, including additional influential factors such as daily dosage and duration of therapy.

The study protocol was approved by the Ethics Committee of the University of Luebeck, Germany (file number: 16–180), and all the participants gave written informed consent.

## Methods

Forty patients (21 PAI, 19 SAI, mean age 52.7, range 20–76) on conventional GRT (2–3 daily doses, range 8–41 mg HC/d, mean: 25.8 mg/d) completed questionnaires evaluating depressive symptoms (Beck Depression Inventory, BDI [20]), subjective health status (Short Form-36, SF-36 [21] and AddiQoL [22]), quality of sleep (Pittsburgh Sleep Quality Index, PSQI [23]), and daytime sleepiness (Epworth Sleepiness Scale, ESS [24]). The BDI is a 21-item self-report rating inventory that measures characteristic attitudes and symptoms of depression in the previous 2 weeks. The standard cut-off scores are as follows: 0–9 points denote minimal depression, 10–18 points indicate mild depression, 19–29 points indicate moderate depression, and 30–63 points to severe depression [25]. The SF-36 tool is a 36-item, patient-reported survey of patient health. It consists of eight scaled scores, which are the weighted sums of the questions in their section. Each scale is transformed into a 0–100 scale; the lower the score, the more disability [21]. The AddiQol-30 is a self-reported disease-specific QoL questionnaire containing 30 items. In total, a maximum of 120 points can be achieved; a high sum score is connected with a high quality of life [26]. The PSQI is a self-report questionnaire assessing sleep quality over the previous 4 weeks. The measure consists of 19 different items which create 7 components that provide an overall score ranging from 0 to 21. Lower scores denote a healthier sleep quality. A PSQI total score of > 5 is indicative of poor sleep [23]. The Epworth Sleepiness Scale (ESS) is an 8-item self-report measure of excessive daytime sleepiness. Respondents indicate on a four-point scale (0 = never, 3 = high chance) the likelihood that they will “doze off or fall asleep” in eight different situations. Responses are summed to yield a total score from 0 to 24, with higher scores indicating greater sleepiness during common daily activities. Total scores of > 7 have been proposed to indicate excessive daytime sleepiness [24].

The results were compared to those of 20 matched (age, sex, educational background) controls. Furthermore, patients’ results were compared contrasting PAI and SAI patients. Additionally, the impact of daily dosage was analyzed using median split. Other hormonal deficits were substituted adequately where necessary. Basically, both groups showed a similar distribution. Patients with antidepressant medication, regular use of anxiolytics, or other sleep-inducing drugs were excluded. Characteristics and information about participants are presented in Table 1.

**Table 1:** Sociodemographic and disease-related data of all participants

**Variable**		**PAI** ***n* = 21**	**SAI** ***n* = 19**	**Patients in total** ***n* = 40**	**Controls** ***n* = 20**
**Age**	20–29 years	3	2	5	4
	30–39 years	1	2	3	1
	40–49 years	5	4	9	3
	50–59 years	5	4	9	4
	60–69 years	4	3	7	3
	70 + years	3	4	7	5
**Sex**	Female	16	12	28	11
	Male	5	7	12	9
**Job**	Yes	13	14	27	12
	No	8	5	13	8
**Shift work**	Yes	0	1	1	1
	No	21	18	39	19
**Education**	Secondary school until 9^th^ class	2	3	5	4
	Secondary school until 10^th^ class	9	8	17	8
	University -entrance	10	8	18	8
**Therapy duration**	6–11 months	1	6	7	
	1–5 years	6	8	14	
	6–10 years	0	2	2	
	11–20 years	6	3	9	
	> 20 years	8	0	8	
**Body mass index (kg/m**^**2**^**)**	< 18.5	1	0	1	
	18.5–24.9	10	8	18	
	25–29.9	9	5	14	
	≥ 30	1	6	7	
**Glucocorticoid**	Hydrocortisone	21	19	40	
	Plenadren	1	1	2	
**Daily dosage (mg)**	≤ 10	0	1	1	
	11–20	7	9	16	
	21–30	9	6	15	
	> 30	5	3	8	
**Dosage schedule**	Twice daily	10	13	23	
	Three times a day	11	6	17	
**Additional substituted endocrine deficits**	Thyroxine	11	12	23	
	Sex hormones	4	3	7	
	Mineralocorticoids	21	0	21	
	Antidiuretic hormone	0	2	2	
	Growth hormone	0	2	2	
	Insulin	1	1	2	

*PAI* primary adrenal insufficiency, *SAI* secondary adrenal insufficiency, *n* number

Data are expressed as mean and standard deviation or as median with range where appropriate (Table 2). Results were compared using the *t* test and the Mann–Whitney *U* test. A *p*-value of < 0.05 was considered significant.

**Table 2:** Results of quality of life and sleep questionnaires

Variable	Patients *n* = 40	Controls *n* = 20	*p*	PAI *n* = 21	SAI *n* = 19	*p*	Short therapy *n* = 20	Long therapy *n* = 20	*p*	Low dosage *n* = 18	High dosage *n* = 22	*p*
BDI	7.0 (23)	2.0 (12)	0.001	9.0 (22)	7.0 (13)	0.253	7.5 (18)	6.0 (22)	0.881	6.5 (16)	7.0 (21)	0.613
SF-36 pss	50.0 (39)	53.6 (28)	0.016	46.0 (26)	52.6 (39)	0.285	52.1 (39)	46.3 (29)	0.176	49.0 (32)	50.0 (32)	0.321
SF-36 mss	43.6 (52)	56.7 (38)	0.001	41.2 (45)	48.7 (52)	0.310	41.3 (52)	47.0 (48)	0.387	52.2 (42)	34.7 (52)	0.034
AddiQoL	82.5 (11.7)	99.9 (11.5)	0.000	78.7 (11.2)	86.7 (10.9)	0.028	82.3 (12.0)	82.7 (11.6)	0.905	84.2 (12.4)	81.1 (11.1)	0.399
PSQI	5.5 (17)	3.0 (19)	0.003	5.0 (17)	6.0 (13)	0.785	5.0 (13)	7.8 (17)	0.288	7.0 (14)	5.0 (16)	0.826
ESS	9.0 (4.3)	6.0 (3.6)	0.009	8.7 (4.5)	9.3 (4.2)	0.640	9.8 (4.6)	8.2 (4.0)	0.230	9.1 (4.2)	8.9 (4.5)	0.858

*BDI* Beck Depression Inventory, *SF-36 pss* short form-36 physical sum scale, *SF-36 mss* short form-36 mental sum scale, *PSQI* Pittsburgh Sleep Quality Index, *ESS* Epworth Sleepiness Scale, *n* number, *PAI* primary adrenal insufficiency, *SAI* secondary adrenal insufficiency

## Results

Although patients with AI scored significantly higher on the Beck Depression Inventory than controls (BDI median 7.0 vs. 2.0; *p* = 0.001), clinically relevant scores were not reached. The rating of the inventory did not reveal depression in either group.

In the SF-36 AI, patients differed significantly from healthy controls and scored less for physical (SF-36 physical sum scale median 50.0 vs. 53.6; *p* = 0.016) and mental health (SF-36 mental sum scale median 43.6 vs. 56.7; *p* = 0.001) than controls; their scores did not reveal substantial impaired functions. However, patients assessed general health (AddiQoL mean: 82.5 vs. 99.9; *p* = 0.000) significantly worse than controls.

The PSQI of patients indicated slightly compromised sleep (PSQI median 5.5 vs. 3.0; *p* = 0.003) and more daytime sleepiness (ESS mean 9.0 vs. 6.0; *p* = 0.009). In particular, patients with HC dosage of > 24 mg/d rated mental health worse (SF-36 mental sum scale median 34.7 vs. 52.2; *p* = 0.034) whereas no negative impact of the daily dose was shown on quality of sleep or daytime sleepiness. Interestingly, there were no significant differences found between patients with PAI and SAI, except in general health assessed by the AddiQoL (mean 78.7 vs. 86.7; *p* = 0.028). Analysis of co-medication showed that only four patients took DHEA. Therefore, further subgroup analysis was not feasible. Duration of therapy did not provoke any significant differences in the variables targeted.

## Discussion

Evaluated data show that patients with AI may suffer from impaired QoL and sleep and therefore confirm and strengthen existing knowledge. However, our present data provide first evidence that patients with PAI and SAI are affected to the same degree, which indicates that the lack of cortisol is a major determinant besides other influential factors like additional hormonal imbalances. Supporting this hypothesis may also be that patients with higher dosage reached fewer points especially in the PSQI, which indicates healthier sleep. Nonetheless, these findings showed no significance, most likely caused by the small number of patients tested. Due to the short half-life of HC, cortisol levels during the night are very low in contrast to the physiological rise in the early morning. Nocturnal HC replacement mimicking this pattern has been analyzed before; however, data concerning the impact on sleep are still missing [27]. Cortisol is known to play a key part in initiating and maintaining different sleep stages. Part of this process is a complex interaction with other mediating substances like melatonin, adrenocorticotropic hormone, and growth hormone-releasing hormone. HC replacement therapy cannot fully restore this cooperation, balance nocturnal hypocortisolism, or normalize sleep architecture [13].

Sleep disturbances in healthy adolescents are associated with increased cardiovascular risk and impaired QoL [28, 29]. Consequently poor sleep quality might be an additional cardiovascular risk factor for patients with AI, facilitating already existing premature mortality. Considering that sleep also determinates QoL and compromised sleep is associated with severe impairments in QoL, which might be even mediated by mental illness [30, 31], quality of sleep should be taken more into account in modern GRT.

The fact that patients with PAI showed worse results in AddiQoL than those with SAI is plausible due to the disease-specific design of this questionnaire [22, 26]. In addition, patients with SAI often have some small ACTH-independent residual function of adrenal cortisol secretion [32].

Our study also showed significantly higher scores in the BDI (but still in the normal range) in the examined collective of AI patients in contrast to controls. In this context, it may be interesting that patients with AI often suffer from psychiatric disorders [33, 34]. Causal research is, however, challenging due to frequent co-morbidity with other endocrine disorders [35] which are also associated with a cumulative occurrence of affective disorders [36].

The present study also demonstrated a negative correlation between high HC dosages and QoL, which supports previous findings [11] but contradict Buning et al. [37]. Liu et al. suggest that the function of the hypothalamus–pituitary–adrenal-axis is intensified in patients with depression, which could strengthen the idea of increased mood disorders in patients with iatrogenic hypercortisolism [38]. Nevertheless, a clear causality between HC dosage and reduced QoL cannot be established, as patients on higher doses may often suffer from ongoing symptoms.

Moreover, our data should be interpreted with caution due to the limited number of patients and the heterogeneity within the group of patients with adrenal insufficiency. Interestingly, there seem to be some AI patients with normal or near-normal self-assessed QoL and sleep in spite of unphysiological replacement. Therefore, further studies should especially focus on the evaluation of subgroups (e.g., patients on higher HC doses or DHEA) who presented with more serious self-rated impairment here and might benefit from replacement modifications.

To sum up, well-conducted substitution therapy should not have any side effects, but the present study emphasizes and expands findings of impaired quality of life, especially concerning general health and poor subjective quality of sleep. The cause is most probably a multidimensional interaction of dysfunctional hormone axes, disrupted sleep architecture and unphysiological GRT, and the reciprocal influence all of these factors should be borne in mind [39]. The present data highlight a gain in importance of psychological care and improvement of sleep to reduce cardiovascular risk and premature mortality and coincidently enhance QoL. Dual-release HC preparations need further research to verify whether they lead to improvements in this context.
